# Usefulness of risk scores and predictors of atrial fibrillation recurrence after elective electrical cardioversion

**DOI:** 10.1111/anec.13095

**Published:** 2023-11-20

**Authors:** Daniel Águila‐Gordo, Javier Jiménez‐Díaz, Martín Negreira‐Caamaño, Jorge Martínez‐Del Rio, Cristina Ruiz‐Pastor, Ignacio Sánchez Pérez, Jesús Piqueras‐Flores

**Affiliations:** ^1^ Cardiology Department Hospital General Universitario de Ciudad Real Ciudad Real Spain; ^2^ Arrhythmia Unit, Cardiology Department Hospital General Universitario de Ciudad Real Ciudad Real Spain; ^3^ Medicine Faculty Castilla La‐Mancha University Ciudad Real Spain; ^4^ Hemodynamics and Interventional Cardiology Unit, Cardiology Department Hospital General Universitario de Ciudad Real Ciudad Real Spain; ^5^ Cardiomyopathies and Inherited Disease Unit, Cardiology Department Hospital General Universitario de Ciudad Real Ciudad Real Spain

**Keywords:** atrial fibrillation recurrence, electrical cardioversion, SLAC score

## Abstract

**Introduction:**

Electrical cardioversion (ECV) is a frequently used procedure for restoring sinus rhythm in atrial fibrillation (AF); however, the rate of recurrence is high. The identification of patients at high risk of recurrence could influence the decision‐making process. The present study evaluates the predictive value of risk scores in atrial fibrillation recurrence after elective electrical cardioversion.

**Methods:**

Unicentric, observational, and prospective study of adult patients who have undergone an elective ECV as rhythm control strategy between July 2017 and September 2022.

**Results:**

From the 283 analyzed patients (mean age 63.95 ± 10.76212, 74.9% male); 99 had paroxysmal AF (35%) and 159 (59%) presented AF recurrence during a follow‐up of 6 months. In patients with post‐ECV AF recurrence, the period of time from diagnosis until the performance of the procedure was longer (393 ± 891 vs. 195 ± 527, *p* = .02). No paroxysmal AF (71.3% vs. 57.8%, *p* = .02) and LA dilatation with >40 mL/m^2^ (35.9% vs. 23.3%, *p* = .02) volumes were more frequent within these patients. AF recurrence was more frequent in patients who had previous ECV (HR = 1.32; 95% CI: 1.12–2.35; *p* = .01) and more than 1 shock to recover sinus rhythm (HR = 1.62; 95% CI: 1.07–1.63; *p* = .01). The SLAC, ALARMEc, ATLAS, and CAAP‐AF scores were statistically significant, although with a moderate predictive capacity for post‐ECV recurrence.

**Conclusions:**

Risk scores analyzed showed a modest value predicting AF recurrence after ECV. Previous ECV, and greater difficulty in restoring SR were independent predictors of recurrence.

## Graphical abstract

The identification of patients at high risk of recurrence could influence the decision‐making process. The risk scores analyzed in the present study showed a modest predictive capacity for AF recurrence after ECV. Time in AF, previous CVE, and a greater difficulty in restoring SR were predictors of recurrence.

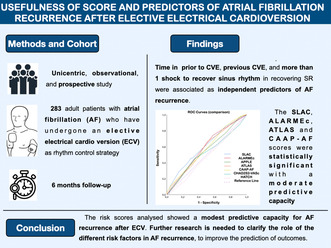

## 1 INTRODUCTION

Atrial fibrillation (AF) is the most frequent arrhythmia in adults and is associated with a significant morbidity and mortality, and a decrease in quality of life (Brandes et al., 2020; Hindricks et al., 2020). AF patients display a higher risk of stroke and almost twofold risk of mortality, compared to the general population (Hindricks et al., 2020; Thangjui et al., 2022). Although rhythm control had been classically considered not to bring any benefit regarding morbidity and mortality (Hagens et al., 2005; Wyse et al., 2002), recent studies highlight the relationship between a higher rate of adverse events and AF progression. Moreover, an early rhythm control strategy has demonstrated to decrease the appearance of cardiovascular events during follow‐up (Willems et al., 2022).

Electrical cardioversion (ECV) is frequently used to control rhythm in AF, with an efficacy rate around 90% and a low percentage of adverse events (Pisters et al., 2012). Nonetheless, the recurrence of AF is common, with rates reaching up to 21% within the first month after the procedure to 60% in the following months (Nuñez‐Garcia et al., 2022; Thangjui et al., 2022; Vitali et al., 2019). Despite several AF relapse predictors being identified, such as non‐paroxysmal pattern, prolonged evolution time, chronic obstructive pulmonary disease (COPD), advanced age, or left atrium (LA) enlargement, the assessment of the individual risk of recurrence is still a challenge as there is no isolated parameter that allows us to reliably predict recurrence. (Pisters et al., 2012; Vizzardi et al., 2014).

The comprehensive approach to elective ECV is complex. The identification of patients at higher risk of post‐ECV AF recurrence could influence the decision‐making process. A better determination of which patients are at high risk of recurrence could help optimize the selection of patients who would benefit the most and allow a modification of the therapeutic strategy through the use of early catheter ablation (CA) and antiarrhythmic drugs.

Over last years, diverse scores have been proposed in order to predict the risk of AF recurrence after a CA procedure (Kornej et al., 2014, 2015; Mesquita et al., 2018; Winkle et al., 2016; Wójcik et al., 2013) in spite of their predictive capacity not being evaluated in elective‐ECV patients yet. In addition, the SLAC score developed specifically for the prediction of recurrence after ECV was subsequently published, but it has not been externally validated yet (3).

The main objective of the study was to analyze if risk scores proposed in CA have a similar predictive value when used to predict recurrence after ECV; as well as to compare them with SLAC score, which has been specifically proposed for this group of patients.

## 2 METHODS

### 2.1 Study design and patient selection

It is a unicentric, observational, and prospective study including a cohort of adult patients with AF detected by surface electrocardiogram (ECG) who underwent an elective ECV as rhythm control strategy between July 2017 and September 2022 in the Hospital General Universitario of Ciudad Real, Spain.

Patients older than 18‐year‐old proposed to undergo ECV were included. The exclusion criteria included spontaneous recovery of sinus rhythm (SR), thrombus identified through transesophageal echocardiogram, ineffective ECV, uncontrolled thyroid disorders, non‐corrected electrolyte imbalances, and incomplete follow‐up.

Assuming an initial recurrence rate of 21%, as previous studies reported (Vitali et al., 2019), and taking into account the possible follow‐up losses, the sample size was estimated for a 0.05 error type I and 0.20 error type II probability.

The study was approved by the Hospital Ethics Committee, informed consent was obtained for data use, and confidentiality was guaranteed at all times, according to the Law of Personal Data Protection and Guarantee of Digital Rights, by means of the development of a decoupled, anonymized database.

### 2.2 Data collection and recurrence score calculation

Clinical history was obtained at the admission to perform the procedure. Data regarding demographic and clinical variables, risk factors, comorbidities, and use of antiarrhythmic drugs were collected. AF classification was done according to current guidelines: paroxysmal AF if there is spontaneous resolution or within the first 7 days thanks to an intervention; persistent AF if it lasts longer than 7 days, including episodes ending up in pharmacological cardioversion (PCV) or ECV; and long‐lasting persistent AF if it lasts longer than 1 year (Hindricks et al., 2020). Prior to ECV, a transthoracic echocardiogram was performed using the Vivid S70 or Vivid E95 (GE Healthcare, Chicago, Illinois, USA) equipment in all patients to quantify the size of the chambers, the left ventricular ejection fraction, and the right ventricular systolic function, as well as to detect the presence of hypertrophy, valve diseases, or pulmonary hypertension, based on current recommendations (Baumgartner et al., 2017; Galderisi et al., 2017; Lancellotti et al., 2013). Blood tests were performed before the procedure.

The CHA_2_DS_2_‐VASc, HATCH, ALARMEc, APPLE, ATLAS, CAAP‐AF, and SLAC scores were calculated for every patient, according to the original definitions (Kornej et al., 2014, 2015; Mesquita et al., 2018; Tang et al., 2012; Thangjui et al., 2022; Winkle et al., 2016; Wójcik et al., 2013). The CHA_2_DS_2_‐VASc score was initially designed to predict the risk of stroke in patients with AF, whereas the HATCH score was developed to stratify the risk of developing persistent AF within 1 year in patients with paroxysmal AF. The ALARMEc, APPLE, ATLAS, and CAAP‐AF scores are currently available to predict post‐CA AF recurrence. On the other hand, the MB‐LATER, BASE‐AF, and CryoAF scores included the early recurrence during the post‐ablation blanking period and, thus, they were not applicable for this work (Mujović et al., 2017). Currently, the SLAC score is the only one that has been developed specifically to assess the risk of recurrence in patients undergoing ECV. Table 1 shows an overall picture of the main risk factors with their corresponding individual scores.

**TABLE 1:** Summary of included risk factors in the different risk scores.

Risk factor	General scores		Specific scores				
	CHA2DS2‐VASc	HATCH	ALARMEc	APPLE	ATLAS	CAAP‐AF	SLAC
Age	✓	✓		✓	✓		
Female sex					✓	✓	
Metabolic syndrome			✓				
AF type			✓	✓	✓	✓	✓
AF duration							✓
LA size			✓	✓	✓	✓	✓
Number of AADs failed						✓	
Current smoking					✓		
HF	✓	✓					
LVEF				✓			
Cardiomyopathy			✓				
Hypertension	✓	✓					
Vascular disease	✓						
Coronary artery disease						✓	
Diabetes Mellitus	✓						
Prior stroke or TIA	✓	✓					
COPD		✓					
Renal dysfunction			✓	✓			
Prior CV							✓

Abbreviations: AAD, antiarrhythmic drug; AF, atrial fibrillation; COPD, chronic obstructive pulmonary disease; CV, cardioversion; HF, heart failure; LA, left atrium; LVEF, left ventricular ejection fraction; TIA, transient ischemic attack.

### 2.3 ECV protocol

An ECV protocol for all the patients included in the study was developed, it was registered and authorized by the Hospital Ethics and Clinical Research Committee. Prior to the performance of the procedure, an ECG was carried out to confirm the diagnosis. The procedures were done using sedation and analgesia with midazolam and etomidate, and with continuous monitoring through disposable self‐adhesive electrodes connected, in anterior–posterior position, to a biphasic defibrillator. The procedure was considered to be successful if it led to restoring the sinus rhythm after the electrical shock. A maximum of 4 shocks with increasing energy were performed: 1st shock 200 Joules (J), 2nd shock 250 J, 3rd shock 300 J, and 4th shock 360 J (maximum energy). After the procedure, patients were required to stay in the Unit until their complete recovery, for a minimum of 6 hours.

### 2.4 Follow‐up

Recurrence was considered if AF (defined by the presence of at least 1 AF episode of more than 30 s in any ECG, 24‐h monitoring, or registry of events in intracardiac devices) was identified. Clinical follow‐up with 12‐lead ECG and 24‐h Holter ECG was regularly carried out after 3 and 6 months. The duration of follow‐up was 6 months. Patients were asked to undergo an ECG, either at the same institution or elsewhere, when feeling palpitations or symptoms of AF recurrence anytime outside monitoring periods, and Holter ECG was added if appropriate. The clinical managers were also warned to inform study staff of the occurrence of cardiac irregularity. In addition, clinical records were reviewed to identify AF episodes during the interim between scheduled visits and, regardless of the reason for their performance, all ECGs performed during follow‐up time were analyzed to identify asymptomatic episodes of atrial fibrillation. Whenever clinical records were insufficient, a structured telephonic interview was conducted.

### 2.5 Statistical analysis

All statistical analyses were carried out using the SPSS software (Version 26.0, IBM, Armonk, New York, USA). Qualitative variables were presented as frequencies and percentages, and quantitative variables as central tendency (median or mean) or dispersion (typical or interquartile range – IQR–) statistical values. In order to compare dichotomous and polytomous variables, the chi‐square test was used; if the expected values of at least 80% of the cells in a contingency table were lower than 5, Fisher's exact test was used. For quantitative variables, distribution normality was assessed by means of the Kolmogorov‐Smirnov test; in case of normal distributions, Student's *t*‐test was used for independent samples, and, in case of variables not following a normal distribution, Mann‐Whitney's *U*‐test was used.

Time‐to‐event analysis was done using Kaplan‐Meier's curves with the log‐rank test. Receiver operating characteristic (ROC) curves of the score systems were created to predict the rhythm results analyzed, and area under the curve (AUC) was used to determine their predictive value. C‐indexes (AUC in ROC) of the analyzed scores were compared using DeLong's method.

For all comparisons, a 5% alpha risk (assuming a statistical significance if *p* < .05) was selected. All intervals show 95% confidence.

## 3 RESULTS

### 3.1 Study population

**FIGURE 1:**
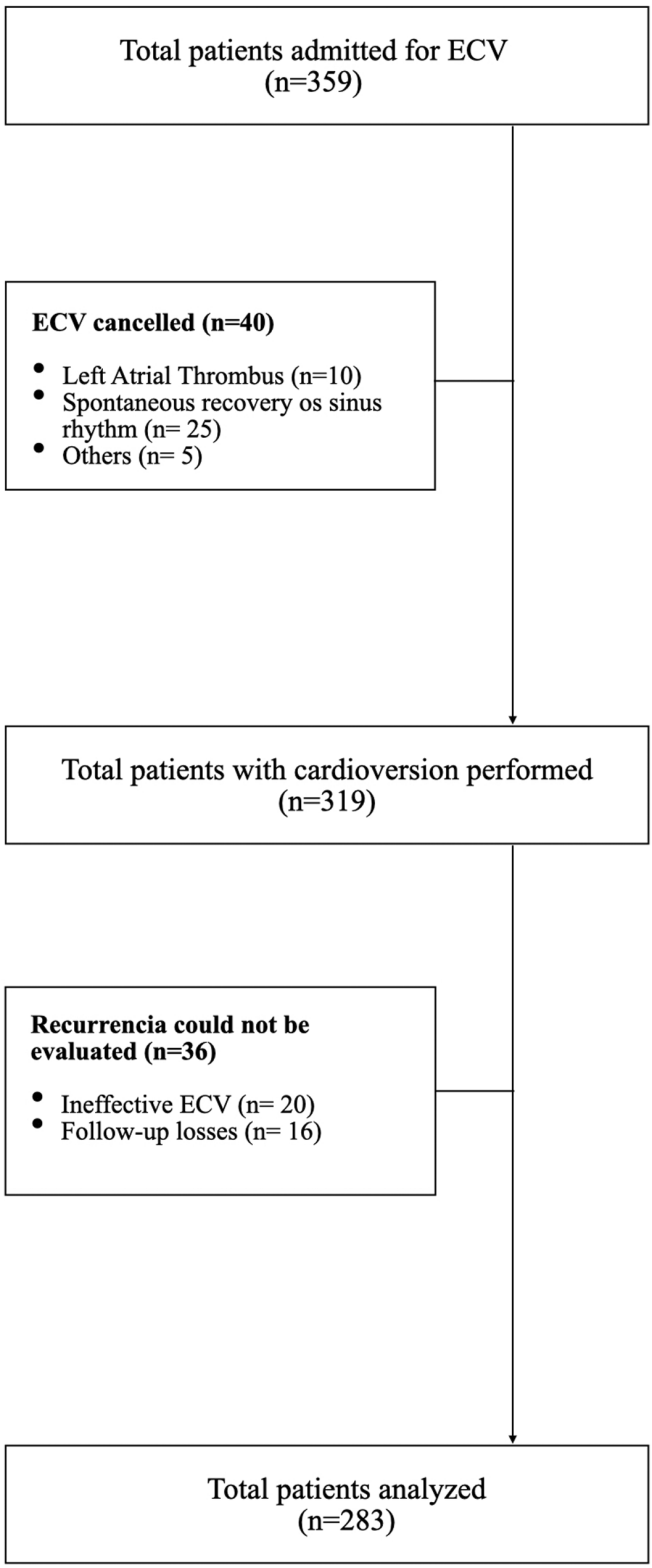
Flow diagram. ECV (electrical cardioversion).

During the period of study, 359 patients with AF, confirmed through ECG, were admitted to undergo elective ECV as rhythm control strategy. In 40 patients, the performance of the procedure was canceled due to spontaneous recovery of the rhythm (*n* = 25), thrombus identified through transesophageal echocardiogram (*n* = 10), or uncontrolled thyroid disorders or non‐corrected electrolyte imbalances (*n* = 5). In 36 patients, the recurrences could not be evaluated due to ineffective ECV (*n* = 20) or incomplete follow‐up (*n* = 16). Finally, 283 patients were analyzed. Figure 1 shows the flow chart.

The mean age of these 283 patients was 63.95 ± 10.76 (range 22–92 years), with 212 patients (74.9%) being male. Paroxysmal, persistent, and long‐lasting persistent AF was detected in 99 (35%), 156 (55.1%), and 28 (9.9%) patients, respectively. The main comorbidities were high blood pressure (66.8%), obesity (47.3%), dyslipidemia (42%), diabetes (19.8%), and heart failure (18.4%). Regarding treatment, 37.1% of patients were on Class III antiarrhythmic drugs, and 11% on Class Ic. Finally, 22.3% of patients had previously undergone an ECV. The basal characteristics of the study population are summarized in Table 2.

**TABLE 2:** (A) Baseline clinical characteristics of the study cohort. (B) Characteristics of cardioversion procedure.

	All patients (*n* = 283)	Patients without AF recurrence (*n* = 116)	Patients with AF recurrence (*n* = 167)	*p*‐Value
(A)				
Age (years ± SD)	63.9 ± 10.76	63.72 ± 10.5	64.11 ± 10.9	.35
Male sex	212 (74.9%)	87 (75%)	124 (74.9%)	.97
Days since AF diagnosis (mean ± SD)	312 ± 767	195 ± 527	393 ± 891	**.02**
≥ 180 days (6 months) since AF diagnosis				
No paroxysmal AF	186 (65.7%)	67 (57.8%)	119 (71.3%)	**.02**
Hypertension	189 (66.8%)	78 (67.2%)	111 (66.5%)	.89
Diabetes Mellitus	56 (19.8%)	23 (19.8%)	33 (19.8%)	.98
Dyslipidemia	119 (42%)	47 (40.5%)	72 (43.1%)	.66
Coronary disease	29 (10.2%)	13 (11.2%)	16 (9.6%)	.65
Prior stroke or TIA	13 (4.6%)	5 (4.3%)	8 (4.8%)	.85
COPD	14 (4.9%)	6 (5.2%)	8 (4.8%)	.88
Current smoking	30 (10.6%)	10 (8.6%)	20 (12%)	.37
OSA	17 (6%)	18 (15.5)	24 (14.4%)	.79
Congestive HF	52 (18.4%)	27 (27.6%)	25 (15%)	.08
CKD (stage 3 and above)	17 (6%)	8 (6.9%)	9 (5.4%)	.61
Obesity (BMI >30)	134 (47.3%)	58 (53.7%)	76 (48.7%)	.42
Previous electrical cardioversion	59 (21.0%)	20 (17.5%)	42 (23.4%)	.16
No. of AADs failed	0.5 ± 0.72	0.43 ± 0.62	0.62 ± 0.77	**.02**
I class AADs	31 (11%)	8 (6.9%)	23 (13.8%)	.07
III class AADs	105 (37.1%)	36 (31.3%)	69 (41.8%)	.74
LAVI >40 mL/m^2^	87 (30.7%)	27 (23.3%)	60 (35.9%)	**.02**
LA diameter > 43 mm	201 (71%)	78 (67.2%)	123 (73.7%)	.24
LVEF <50%	85 (30%)	38 8(32.8%)	47 (28.1%)	.41
Valvular heart disease moderate or severe	58 (24.5%)	24 (25%)	58 (24.5%)	.87
(B)				
AAD facilitated ECV	102 (36.2%)	38 (32.8%)	64 (38.6%)	.32
Post‐ECV AADs	242 (85.5%)	103 (88.8%)	139 (83.2%)	.19
>1 shock	52 (18.6%)	14 (12.3%)	38 (22.9%)	**.03**
Maximum energy (360 J) shocks	13 (4.6%)	3 (2.6%)	10 (6.1%)	.17
Minor complications	17 (6.0%)	9 (7.8%)	8 (4.8%)	.31
Major complications	3 (1.1%)	1 (0.9%)	2 (1.2%)	.78

*Note*: All values are mean ± _SD for continuous variables and number (%) for categorical variables. Current smoking was defined as any cigarette consumption during the previous 6 months to ECV.

Bold values for *p*‐value < .05.

Abbreviations: AAD, antiarrhythmic drugs; AF, atrial fibrillation; BMI, body mass index; CKD, Chronic kidney disease; COPD, chronic obstructive pulmonary disease; ECV, electrical cardioversion; GFR, glomerular filtration rate; HF, heart failure; LA, left atrium; LAVI, left atrial volume index (mL/m^2^); LVEF, left ventricular ejection fraction; OSA, obstructive sleep apnea; SD, standard deviation; TIA, transient ischemic attack.

### 3.2 Data related to the procedure

The mean number of shocks administered was 1.26 ± 0.66; with an average energy of 288 ± 179.3 J. The adverse events were scarce: 6% developed minor adverse events (burns, skin irritation, or non‐extreme bradycardia), whereas 1.1% developed major complications (stroke, tachycardia, ventricular fibrillation, or extreme bradycardia). There was only 1 patient (0.4%) who suffered a stroke after the ECV. A total of 36.5% of ECVs were facilitated by antiarrhythmic drugs: 89.5% with amiodarone, 4.8% with Ic antiarrhythmics, and 3.8% with sotalol. The characteristics of the procedure are displayed in Table 2B.

### 3.3 Post‐ECV AF recurrence

**FIGURE 2:**
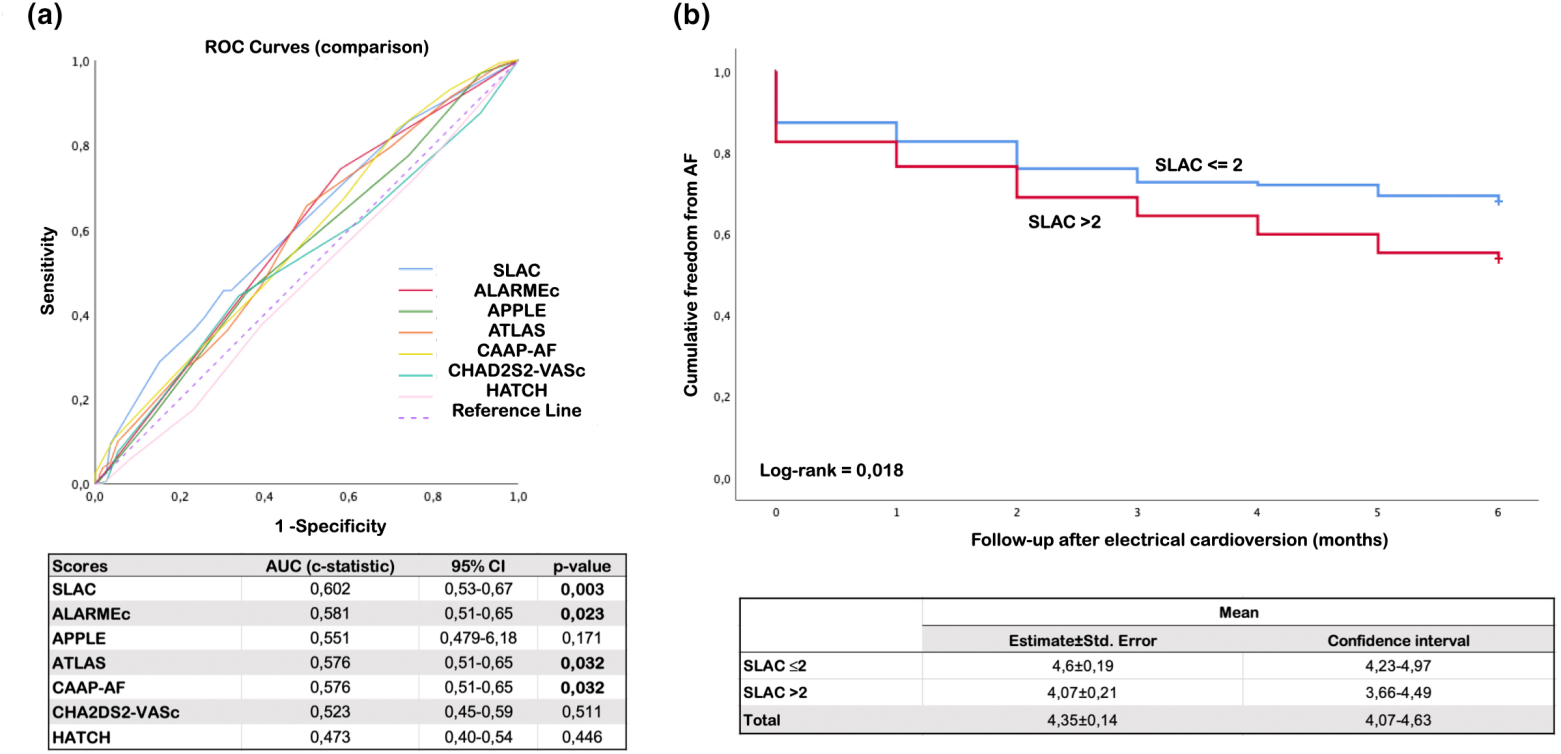
(a) Receiver Operating Characteristic (ROC) curve analysis of tested scores for prediction of atrial fibrillation (AF) recurrence after electrical cardioversion (ECV). (b) Kaplan‐Meier curve of AF‐free survival after ECV according to cut‐off value of SLAC score (≤2or >2).

AF recurrence was detected in 159 patients (59%). In patients with post‐ECV AF recurrence, the period of time from diagnosis until the performance of the procedure was longer (393 ± 891 days vs. 195 ± 527 days, *p* = .02). Paroxysmal AF (57.8% vs. 71.3%, *p* = .02) or LA dilatation with <40 mL/m^2^ volumes (23.3% vs. 35.9%, *p* = .02) were less frequent among these patients. More than 1 shock to obtain an effective ECV (22.9% vs. 12.3%, *p* = .03) was associated with a higher incidence of recurrence during follow‐up. Patients with a successful ECV after the administration of the first shock showed a lower recurrence during follow‐up (56.5% vs. 87%, *p* = .06). Patients with AF recurrence showed higher levels of NT‐proBNP (1153 ± 1548 pg/mL vs. 358 ± 479 pg/mL, *p* = .01) with no differences in the other parameters analyzed (Figure 2).

Univariate (crude) and multivariate (adjusted) Cox regression models were performed for baseline characteristics of the study population and the procedure (Table 3). In the adjusted model, a higher risk of earlier recurrence was observed patients who had previously undergone ECV (HR = 1.32; 95% CI: 1.12–2.35; *p* = .01) and those who had required more than one shock to recover SR (HR = 1.61; 95% CI: 1.11–2.32; *p* = .01).

**TABLE 3:** (A) Cox regression. Baseline clinical characteristics of the study cohort. (B) Cox regression. Characteristics of cardioversion procedure.

	Univariate analysis			Multivariate analysis		
	HR	95% CI	*p*‐Value	HR	95% CI	*p*‐Value
(A)						
Age (years ± SD)	0.99	0.98–1.01	.86	1.02	0.98–1.02	.84
Male sex	0.94	0.66–1.34	.75	1.06	0.73–1.54	.75
Days since AF diagnosis (mean ± SD)	1.01	1.01–1.02	**.01**	1.01	0.99–1.03	.11
No paroxysmal AF	0.95	0.69–1.31	.77			
Hypertension	0.87	0.6–1.28	.51			
Diabetes Mellitus	1.24	0.93–1.73	.14			
Dyslipidemia	1.01	0.61–1.69	.97			
Coronary disease	1.27	0.63–2.6	.51			
Prior stroke or TIA	0.83	0.41–1.7	.62			
COPD	1.06	0.99–1.13	.08			
Current smoking	1.04	0.67–1.6	.86			
OSA	0.82	0.54–1.3	.36			
Congestive HF	0.97	0.49–1.91	.94			
CKD (stage 3 and above)	0.91	0.66–1.25	.57			
Obesity (BMI >30)	1.2	0.86–1.54	.34			
Previous electrical cardioversion	1.58	1.01–2.28	**.01**	1.55	1.03–2,35	**.03**
No. of AADs failed	1.2	0.86–1.54	.34			
I class AADs	1.18	0.76–1.83	.46			
III class AADs	1.1	0.8–1.5	.57			
LAVI >40 mL/m^2^	1.05	0.99–1.11	.06	1.04	0.99–1.1	.09
LA diameter > 43 mm	1.2	0.85–1.7	.3			
LVEF <50%	1.08	0.77–1.51	.66			
Valvular heart disease moderate or severe	1.02	0.69–1.5	.91			
(B)						
AAD facilitated ECV	1.2	0.87–1.65	.25			
Post‐ECV AADs	0.73	0.48–1.1	.13			
>1 shock	1.57	1.09–2.27	**0.01**	1.71	1.18–2.47	**.01**
Maximum energy (360 J) shocks	1.7	0.89–.21	0.11			
Minor complications	0.65	0.32–1.33	.24			
Major complications	4.05	0.98–16.6	0.06			

*Note*: Cox univariate (crude) and multivariate (adjusted) analysis. All values are mean ± _SD for continuous variables and number (%) for categorical variables. Current smoking was defined as any cigarette consumption during the previous 6 months to ECV.

Bold values for *p*‐value < .05.

Abbreviations: AAD, antiarrhythmic drugs; AF, atrial fibrillation; BMI, body mass index; CKD, Chronic kidney disease; COPD, chronic obstructive pulmonary disease; ECV, electrical cardioversion; GFR, glomerular filtration rate; HF, heart failure; LA, left atrium; LAVI, left atrial volume index (mL/m^2^); LVEF, left ventricular ejection fraction; OSA, obstructive sleep apnea; SD, standard deviation; TIA transient ischemic attack.

### 3.4 Evaluation of the AF recurrence risk score

The association between the different elements of the diverse scores and the risk of AF recurrence are reflected in Table S1. Patients with post‐ECV AF recurrence presented a higher SLAC score (3.59 ± 3.21 vs. 2.53 ± 2.83, *p* < .01). The means of the ALARMEc (1.91 ± 0.83 vs. 1.70 ± 0.91, *p* = .04), CAAP‐AF (5.35 ± 1.81 vs. 4.75 ± 1.92, *p* < .01), and SLAC (3.59 ± 3.21 vs. 2.53 ± 2.88, *p* < .01) scores were higher in patients with AF recurrence, although in the unadjusted Cox analysis the ATLAS (HR = 1.05; IC95% = 1.01–1.1) and SLAC (HR = 1.06; IC95% = 1.01–1.11) scores were the ones associated with a higher recurrence risk.

The analysis of the ROC curves of the analyzed scores is displayed in Figure 1a. The SLAC, ALARMEc, ATLAS, and CAAP‐AF scores demonstrated to have a statistically significant predictive capacity. The highest AUC value was found in the SLAC score (AUC = 0.602; IC95% = 0.53–0.67), followed by the ALARMEc (AUC = 0.581; IC95% = 0.51–0.65), ATLAS (AUC = 0.576; IC95% = 0.51–0.65), and CAAP‐AF (AUC = 0.576; IC95% = 0.51–0.65) scores, with no differences between them in the DeLong test.

The SLAC score had the highest AUC value. A SLAC score higher than 2 was identified as the best cut‐off to predict post‐ECV AF recurrence, with 53% sensitivity (95% CI 44.9%–60.5%) and 61.2% specificity (95% CI 51.72%–70.11%). The remaining diagnostic indicators are displayed in Table S2. A Kaplan‐Meier survival analysis was performed to evaluate the time free from AF based on SLAC score, with patients with a ≤2 score having a higher time free from AF than those with >2 scores 2 (median 4.6 ± 0.19 months vs. 4.07 ± 0.21 months; log rank, *p* = .018) (Figure 1b).

The LA size was a predictive factor associated with a higher incidence of recurrence. The LA indexed volume (mL/m^2^), present in the ATLAS score (HR = 1.28; IC = 1.04–1.56; *p* = .04), in which one point was given for each 10 mL/m^2^, was a statistically significant risk factor. Moreover, the atrial size in the SLAC score, in which a volume higher than 40 mL/m^2^ corresponded to 6 points, also showed to be associated with risk of recurrence (HR = 1.05; IC = 0.99–1.11, *p* = .06). The atrial diameter, as evaluated in the CAAP‐AF score was a predictor of recurrence (HR = 1.2; IC = 1.02–1.43). Besides, it is worth highlighting the association between previous ECV and the risk of recurrence, reflected in the SLAC score (HR = 1.05; IC = 1.08–2.2).

Finally, in Table S3, a univariate analysis is reflected combining significant variables of AF recurrence as well as SLAC score (which already includes LA size and prior ECV among its variables). The combination of LAVI >40 mL/m^2^ + Previous ECV+ >1 shock (HR = 1.07; IC = 1.02–1.13) to recover SR was a useful parameter for identifying patients at higher risk of recurrence, whereas combinations that included “days since AF diagnosis” as variables, although statistically significant, were certainly of little clinical relevance due to small HR values.

## 4 DISCUSSION

The present study evaluates the usefulness of AF recurrence risk scores after elective ECV, obtaining a modest predictive capacity, with SLAC score showing the highest performance. On the other hand, previous ECV, and greater difficulty in recovering SR were associated as independent predictors of AF recurrence. In addition, LA volume > 40 mL/m^2^ and longer time in AF before ECV were more frequent in this group of patients.

AF is a progressive and evolving disease that can progress from self‐limited paroxysms to persistent and long‐lasting forms. This progression is usually associated with electrical and structural changes in LA. For instance, LA dilatation creates a feedback mechanism by promoting remodeling and perpetuating the causative mechanisms of AF (Eckstein et al., 2008). As expected, one of the main predictors of recurrence of AF was LA size at the time of ECV, as well as the episode duration and burden of AF.

The atrial size can be measured as a diameter, an area, or a volume, with LA indexed volume being the most accurate measure to evaluate atrial asymmetry (Lang et al., 2015). The LA volume measured by transthoracic echocardiography may be superior to LA diameter in predicting adverse outcomes, including recurrent AF (Kranert et al., 2020; Marchese et al., 2012). In this sense, the findings obtained in our study reinforce this statement, since LA volume > 40 mL/m^2^ was more prevalent in the recurrence group and showed a trend toward significance in survival analysis. In this regard, SLAC score that considers LA indexed volume as one of its variables is accurate and probably is one of the reasons it shows better predictive capacity.

This study highlights the prognostic impact of previously attempted cardioversion. As in other studies, prior history of ECV was an important independent predictor of recurrence (Boriani et al., 2007; Martínez‐Brotóns et al., 2006; Thangjui et al., 2022). This finding suggests that ECV may be worthy in the absence of previous attempts and other predictors of recurrence, with the aim to provide these patients with a chance of restoring SR (Boriani et al., 2007). Unlike atrial size, which is included in all risk scores analyzed, only SLAC score considers previous cardioversion attempts as one of its variables, which may contribute to increase its performance.

Furthermore, difficulty in restoring SR is a powerful predictor of recurrence, thus, failed attempts of ECV and a higher number of shocks have been associated with a higher recurrence rate (Alla et al., 2012; Pisters et al., 2012). However, unlike recurrence predictors previously described, number of shocks during ECV has not been considered in SLAC score.

The clinical predictive power of these recurrence factors may be limited and probably insufficient to abandon the rhythm control strategy in patients who could benefit from preserving the sinus rhythm (Jaakkola et al., 2017; Raitt et al., 2006; Zohar et al., 2005). For this reason, risk scores could be simple, useful, and appealing tools to assist physicians in predicting the recurrence risk. Even though CHA2DS2‐VASc and HATCH risk scores were originally designed for different objectives, other than predicting AF recurrence, both have been validated for this purpose in patients undergoing CA and ECV. Each component of those scores separately, which are basically cardiovascular risk factors and age, have shown a low discriminative capacity as they do not include important predictors of recurrence such as atrial size, evolution time or AF pattern (Mulder et al., 2021; Pisters et al., 2012; Vitali et al., 2019). In alignment with the available evidence, in our study, neither CHA2DS2‐VASc nor HATCH scores showed discriminative capacity or statistically significant difference in ROC curves analysis.

In recent years, several specific risk scores have been designed to predict AF recurrence after CA; however, they were not properly evaluated in patients undergoing ECV. The different scores analyzed in our study showed limited predictive value for prognosticating recurrences in patients undergoing ECV. The ATLAS and APPLE scores assessed in patients undergoing ECV by Thangjui et al showed similar results to our study (Thangjui et al., 2022). Applied to patients undergoing CA, their predictive capacity has been only slightly superior to that shown in ECV, so future research is needed to clarify their real potential and usefulness (Dretzke et al., 2020).

The SLAC score has recently been published as the first scoring system specifically designed to predict AF recurrence after elective ECV and includes as variables the AF pattern, LA volume > 40 mL/m^2^, previous ECV and history of stroke or transient ischemic attack (TIA) (Thangjui et al., 2022). In our study, the first three variables were associated with higher recurrence during follow‐up, in contrast with previous history of stroke/TIA, which had a neutral impact. The SLAC score was the one that obtained a better performance in terms of better discriminative capacity and predictive value. In the original cohort, patients were classified into three risk groups, low risk (0–2 points), moderate risk (3–8 points), and high risk (9–14 points) (Thangjui et al., 2022) and the sensitivity and specificity for patients with >3 points was 81.7% and 60.7%. Nevertheless, in the present study the score ≥2 points was the cut‐off point with better sensitivity and specificity values for predicting AF recurrence, although they were significantly lower than in the original cohort (Thangjui et al., 2022). Although post‐ECV follow‐up and recurrence rate were similar in both cohorts, the baseline characteristics, including antiarrhythmic drugs were different, which was expected given the heterogeneous nature of AF and may account for the lower diagnostic power obtained.

In the present study, the discriminative value of the evaluated scores is modest, limiting the use as a unique tool in clinical practice to detect patients in whom ECV might be futile. In addition, it should be also noted that there are situations in which ECV could work not only as a “therapeutic” option but also as a differential diagnostic tool in some patients with persistent AF, such as those with heart failure, as the relationship between symptoms and arrhythmia might be unclear. For that reason, some authors suggest that a “diagnostic ECV” could be performed to clarify whether the symptoms of the patients improve or not after ECV in sinus rhythm, and, if so, this could lead to the performance of a more invasive strategy to preserve sinus rhythm, such as CA (Brandes et al., 2020). In this group of patients, the predictive value of recurrence risk scores is less useful, given that in this case the aim is to assess the clinical response to restoration of SR, regardless of arrhythmia recurrence.

However, predicting AF recurrence after ECV is a practical clinical goal and may require a more comprehensive and complex approach to achieve it. Artificial intelligence models are currently being developed with the aim of improving the accuracy of predictions of restoration and maintenance of SR (Nuñez‐Garcia et al., 2022).

The main limitations of our study are the small sample size and its unicentric nature. Additionally, the post‐ECV follow‐up and rhythm monitoring were intermittent, which could have led to missing some recurrence cases if those would have been asymptomatic episodes. Nonetheless, our follow‐up is similar to the follow‐up reported in previous studies, which also did not include continuous ECG monitoring (Kornej et al., 2015; Mesquita et al., 2018; Mujović et al., 2017; Thangjui et al., 2022; Winkle et al., 2016). In spite of the existence of other clinical scores to predict post‐CA and post‐PCV recurrence, they could not be evaluated as we did not have the necessary data.

## 5 CONCLUSION

The risk scores analyzed in the present study showed a modest predictive capacity for AF recurrence after ECV, obtaining a modest predictive capacity, with SLAC score showing the highest performance. On the other hand, previous ECV and greater difficulty in recovering SR were associated as independent predictors of AF recurrence. In addition, LA volume > 40 mL/m^2^ and longer time in AF before were more frequent in this group of patients. Nevertheless, further research is needed to clarify the role of the different risk scores and factors in AF recurrence, to improve the prediction of outcomes.

## AUTHOR CONTRIBUTIONS

DAG, JJD, JPF: design, data collection, analysis, writing, review. MNC, JMR, CRP data collection and review. ISP: review.

## CONFLICT OF INTEREST STATEMENT

The authors have no conflicts of interest to declare.

## ETHICS STATEMENT

The study was approved by Ethics Committee of the hospital with the code C‐407.

## Supporting information


Appendix S1

## Data Availability

The data that support the findings of this study are available from the corresponding author upon reasonable request.
