# Supplementary material for: Usefulness of risk scores and predictors of atrial fibrillation recurrence after elective electrical cardioversion
Source: Ann Noninvasive Electrocardiol. 2023 Nov 20;29(1):e13095. doi: 10.1111/anec.13095 (PMC10770812; doi:10.1111/anec.13095)
Supplement: Supplementary file 1 — Appendix S1 [file ANEC-29-e13095-s001.docx]

**SUPPLEMENTARY DATA**

**Title:** Usefulness of score and predictors of atrial fibrillation recurrence after elective electrical cardioversion.

**Authors:** Águila-Gordo, Daniel, MD^a^, Jiménez-Díaz, Javier, MD, PhD^b^, Negreira-Caamaño, Martín, MD^a^, Martínez-Del Rio, Jorge MD^a^ Ruiz-Pastor, Cristina^c^, Sánchez Pérez, Ignacio, MD^d^ Piqueras-Flores, Jesús, MD, PhD^de*^

^a^Cardiology Department, Hospital General Universitario de Ciudad Real, Ciudad Real, Spain.

^b^Arrhythmia Unit, Cardiology Department, Hospital General Universitario de Ciudad Real, Ciudad Real, Spain.

^c^Medicine Faculty, Castilla La-Mancha University, Ciudad Real, Spain.

D Hemodynamics and Interventional Cardiology Unit, Cardiology Department, Hospital General Universitario de Ciudad Real, Ciudad Real, Spain.

^e^Cardiology Department, University General Hospital of Ciudad Real, Spain; Cardiomyopathies and Inherited Disease Unit, Cardiology Department, Hospital General Universitario de Ciudad Real, Ciudad Real, Spain.

| Table 1. Components of different clinical scores in relation to AF recurrence | | | | | | | | | |
| --- | --- | --- | --- | --- | --- | --- | --- | --- | --- |
|  |  | | | |  | Univariate analysis | | | |
| Characteristics | All patients  (n= 283) | Patients without AF recurrence (n=116) | | Patients without AF recurrence (n=167) | p-value | | HR | 95% CI | p-value |
| **ALARMAEc** | 1.82±0.87 | 1.70±0.91 | 1.91±0.83 | | **0.04** | 1.1 | | 0.93-1.32 | 0.26 |
| No paroxymal AF | 186 (65.7%) | 67 (57.8%) | 119 (71.3%) | | **0.02** | 1.34 | | 0.96-1.86 | 0.08 |
| NLA>11.5 | 219 (77.9%) | 86 (74.8%) | 133 (80.1%) | | 0.29 | 1.15 | | 0.78-1.68 | 0.48 |
| GFR < 68 mL/min/1.73 m2 | 61 (21.6%) | 24 (20.7%) | 37 (22.25%) | | 0.76 | 0.98 | | 0.68-1.42 | 0.91 |
| Metabolic syndrome | 44 (15.5%) | 16 (13.8%) | 28 (16.8%) | | 0.49 | 1.06 | | 0.7-1.58 | 0.79 |
| Dilated/hypertrophic cardiomyopathy | 7 (2.5%) | 4 (3.4%) | 3 (1.8%) | | 0.38 | 0.65 | | 0.21-2.08 | 0.47 |
| **APPLE** | 2.28±1.1 | 2.16±1.13 | 2.37±1.07 | | 0.13 | 1.1 | | 0.95-1.26 | 0.22 |
| Age>65 years | 123 (43.6%) | 48 (41.4%) | 76 (45.5%) | | 0.44 | 1.34 | | 0.96-1.86 | 0.08 |
| No paroxymal AF | 186 (65.7%) | 67 (57.8%) | 119 (71.3%) | | **0.02** | 1.34 | | 0.96-1.86 | 0.08 |
| GFR <60 mL/min/1.73 m2 | 52 (18.4%) | 20 (17.2%) | 32 (19.2%) | | 0.68 | 1.01 | | 0.68-1.47 | 0.98 |
| LAD>43 mm | 201 (71%) | 78 (67.2%) | 123 (73.7%) | | 0.24 | 1.21 | | 0.85-1.71 | 0.28 |
| LVEF <50% | 85 (30%) | 47 (28.1%) | 38 (32.8%) | | 0.41 | 1.1 | | 0.78-1.54 | 0.58 |
| **ATLAS** | 7.84±3.04 | 7.43±3.01 | 8.13±3.03 | | **0.06** | 1.05 | | 1.01-1.1 | **0.04** |
| Age>60 years | 180 (63.6%) | 106 (63.5%) | 74 (63.8%) | | 0.96 | 0.99 | | 0.72-1.36 | 0.96 |
| No paroxymal AF | 186 (65.7%) | 67 (57.8%) | 119 (71.3%) | | **0.02** | 1.34 | | 0.96-1.86 | 0.08 |
| LAVI (ml/m2) | 44.15±14.2 | 36.8±5.06 | 46.32±15.37 | | 0.19 | 1.28 | | 1.04-1.56 | **0.02** |
| Female sex | 71 (25.2%) | 29 (25%) | 42 (25.1%) | | 0.9 | 0.98 | | 0.9-1.07 | 0.72 |
| Current smoking | 30 (10.6%) | 10 (8.6%) | 20 (12%) | | 0.37 | 1.06 | | 0.99-1.13 | 0.08 |
| **CAAP-AF** | 5.11±1.87 | 4.75±1.92 | 5.35±1.81 | | **<0.01** | 1.07 | | 0.99-1.16 | 0.09 |
| CAD | 29 (10.2%) | 13 (11.2%) | 16 (9.6%) | | 0.65 | 1.01 | | 0.59-1.68 | 0.99 |
| LAD (mm) | 45.45±6.2 | 44.71±5.6 | 45.86±6.6 | | 0.92 | 1.2 | | 1.02-1.43 | **0.03** |
| Age (years) | 63.9±10.76 | 63.72±10.5 | 64.11±10.9 | | 0.35 | 0.98 | | 0.82-1.15 | 0.76 |
| No paroxymal AF | 186 (65.7%) | 67 (57.8%) | 119 (71.3%) | | **0.02** | 1.34 | | 0.96-1.86 | 0.08 |
| Failed AADs | 0.5±0.72 | 0.43±0.62 | 0.62±0.77 | | **0.02** | 1.14 | | 0.85-1.52 | 0.36 |
| Female sex | 71 (25.2%) | 29 (25.2%) | 42 (25.1%) | | 0.9 | 0.98 | | 0.9-1.07 | 0.72 |
| **CHA2DS2-VASc** | 2.18±1.46 | 2.11±1.42 | 2.23±1.48 | | 0.51 | 1.05 | | 0.93-1.19 | 0.42 |
| Congestive HF | 52 (18.4%) | 27 (23.3%) | 25 (15%) | | 0.08 | 0.82 | | 0.54-1.26 | 0.36 |
| Hypertension | 189 (66.8%) | 78 (67.2%) | 111 (66.5%) | | 0.89 | 0.96 | | 0.69-1.33 | 0.81 |
| Age ≥75 | 51 (18%) | 18 (15.5%) | 33 (19.8%) | | 0.36 | 1.15 | | 0.78-1.69 | 0.48 |
| Diabetes Mellitus | 56 (19.8%) | 33 (19.8%) | 23 (19.8%) | | 0.98 | 0.87 | | 0.59-1.28 | 0.48 |
| Prior stroke or TIA | 13 (4.6%) | 5 (4.3%) | 8 (4.8%) | | 0.85 | 1.26 | | 0.62-2.57 | 0.51 |
| Vascular disease | 48 (17%) | 28 (16.8%) | 20 (17.2%) | | 0.92 | 1.18 | | 0.79-1.78 | 0.41 |
| Age 65-75 | 83 (29.3%) | 35 (30.2%) | 48 (28.7%) | | 0.79 | 0.88 | | 0.63-1.23 | 0.46 |
| Female sex | 71 (25.2%) | 29 (25.2%) | 42 (25.1%) | | 0.9 | 0.98 | | 0.9-1.07 | 0.72 |
| **HATCH** | 1.36±1.23 | 1.43±1.31 | 1.32±1.17 | | 0.44 | 0.94 | | 0.79-1.11 | 0.46 |
| Hypertension | 189 (66.8%) | 78 (67.2%) | 111 (66.5%) | | 0.89 | 0.96 | | 0.69-1.33 | 0.81 |
| Age ≥75 | 51 (18%) | 18 (15.5%) | 33 (19.8%) | | 0.36 | 1.15 | | 0.78-1.69 | 0.48 |
| Prior stroke or TIA | 13 (4.6%) | 5 (4.3%) | 8 (4.8%) | | 0.85 | 1.26 | | 0.62-2.57 | 0.51 |
| COPD | 14 (4.9%) | 6 (5.2%) | 8 (4.8%) | | 0.86 | 0.92 | | 0.45-1.87 | 0.82 |
| Congestive HF | 52 (18.4%) | 27 (23.3%) | 25 (15%) | | 0.08 | 0.82 | | 0.54-1.26 | 0.36 |
| **SLAC** | 3.15±3.12 | 2.53±2.88 | 3.59±3.21 | | **<0.01** | 1.06 | | 1.01-1.11 | **<0.01** |
| No paroxymal AF | 186 (65.7%) | 67 (57.8%) | 119 (71.3%) | | **0.02** | 1.1 | | 0.96-1.25 | 0.1 |
| Prior stroke or TIA | 13 (4.6%) | 5 (4.3%) | 8 (4.8%) | | 0.85 | 1.26 | | 0.62-2.57 | 0.51 |
| Prior CV | 63 (22.3) | 21 (18.1%) | 42 (25.1%) | | 0.16 | 1.54 | | 1.08-2.2 | **0.02** |
| LAVI >40 ml/m2 | 87 (30.9) | 27 (23.5%) | 60 (35.9%) | | **0.02** | 1.05 | | 0.99-1.11 | 0.06 |
| Univariate Cox regression analysis. AF, atrial fibrillation; CAD, coronary artery disease; CI, confidence interval; COPD, chronic obstructive pulmonary disease; CV, cardioversion; Failed AADs, number of refractory antiarrhythmics before cardioversion; GFR; glomerular filtration rate; HF, heart failure; HR, hazard ratio; LAD, left atrium diameter; LAVI, left atrial volume index; LVEF, left ventricular ejection fraction; NLA, normalized left atrial área; TIA, transient ischemic attack. | | | | | | | | | |

| Table 2. SLAC risk score diagnostic test indicators | | |
| --- | --- | --- |
|  | Value | CI 95% |
| Sensitivity | 53.00% | 44.8-60.5 |
| Specificity | 61.20% | 51.7-70.1 |
| Positive predictive value | 66.20% | 59.9-71.9 |
| Negative predictive value | 47.30% | 42-52.7 |
| Positive likehood ratio (LR+) | 1.36 | 1.04-1.78 |
| Negative likehood ratio (LR-) | 0.77 | 0.62-0.96 |

| Table 3. Combination of the predictors of AF recurrence | | | |
| --- | --- | --- | --- |
|  | Univariate analysis | | |
| Combination of 3 predictors | HR | 95% CI | p-value |
| Days since AF diagnosis + LAVI >40 ml/m2 + Previous ECV | 1.002 | 1.002-1.003 | **<0.01** |
| Days since AF diagnosis + LAVI >40 ml/m2 + >1 shock | 1.001 | <1.001-1.002 | **<0.01** |
| Days since AF diagnosis + Previous ECV + >1 shock | 1.002 | 1.002-1.003 | **<0.01** |
| Days since AF diagnosis + SLAC score + >1 shock | 1.001 | <1.001-1.002 | **<0.01** |
| LAVI >40 ml/m2 + Previous ECV+ >1 shock | 1.07 | 1.02-1.13 | **<0.01** |
| Combination of 4 predictors | HR | 95% CI | p-value |
| Days since AF diagnosis + LAVI >40 ml/m2 + Previous ECV + >1 shock | 1.001 | <1.001-1.002 | **<0.01** |
| AF, atrial fibrillation; ECV, electrical cardioversion; LAVI, left atrial volume index (ml/m2). | | | |
